# Progranulin protects against *Clostridioides difficile* infection by enhancing IL-22 production

**DOI:** 10.1080/19490976.2024.2409220

**Published:** 2024-09-30

**Authors:** Jun Huang, Bichen Liu, Yi Liu, Wenxian You, Ping Zhao, Yuhan Liu, Kehan Wang, Xiaofei Lai, Banglao Xu, Ju Cao

**Affiliations:** a Department of Laboratory Medicine, The First Affiliated Hospital of Chongqing Medical University, Chongqing, China; b Department of Laboratory Medicine, Fujian Medical University Union Hospital, Fuzhou, China; c Department of Surgery, School of Medicine, Stanford University, Stanford, USA; d Department of Gastroenterology, The First Affiliated Hospital of Chongqing Medical University, Chongqing, China; e Department of Laboratory Medicine, Guangzhou First People’s Hospital, School of Medicine, South China University of Technology, Guangzhou, Guangdong, China

**Keywords:** *Clostridioides difficile*, infection, progranulin, immunity, interleukin-22

## Abstract

Enhanced mortality, relapse rates, and increased prevalence of *Clostridioides difficile* infection (CDI) emphasize the need for better therapies and management approaches. Modulating host immune response to ameliorate CDI-associated immunopathology may provide new advantages to currently inadequate antibiotic therapies. Here, we identified progranulin (PGRN) as an important immune target upregulated in response to CDI. PGRN-deficient mice displayed dramatically higher mortality and aggravated epithelial barrier disruption compared with wild type (WT) mice after CDI despite equivalent levels of bacterial burden or toxin in the large intestine. Mechanistically, PGRN protection was mediated by IL-22 production from CD4^+^ T helper cells, as demonstrated by a decrease in colonic IL-22-producing CD4^+^ T helper cells in the intestine of PGRN-deficient mice upon CDI and a boost of IL-22-producing CD4^+^ T helper cells activated by PGRN *ex vivo*. Clinical evidence suggests that CDI patients had significantly higher serum levels of PGRN compared with healthy controls, which was significantly and positively correlated with IL-22. Our findings thus indicate a critical role for PGRN-promoted CD4^+^ T cell IL-22 production in shaping gut immunity and reestablishing the intestinal barrier during CDI. As an alternative to pathogen-targeted therapy, this study may provide a new host-directed therapeutic strategy to attenuate severe, refractory CDI.

## Introduction

*Clostridioides difficile* infection (CDI) is currently the most common cause of hospital-acquired diarrhea in many countries and is responsible for substantial morbidity, mortality, and health-care costs.^1^ As a result, *Clostridioides difficile* has been listed as one of the three urgent threats by the Centers for Disease Control and Prevention.^2^ Upon germination, *C. difficile* produces chief virulence factors, toxins A and B. These toxins enter cells via receptor-mediated endocytosis and inactivate small GTPases by glucosylating a key residue, resulting in disruption of the actin cytoskeleton and loss of integrity of the intestinal barrier, which leads to an increase in gut permeability, inflammation, and disease.^3^ CDI ranges from diarrhea to life-threatening pseudomembranous colitis and toxic megacolon.^4^ Therefore, CDI should be promptly treated, or it can quickly progress to fatal disease.

The antibiotics vancomycin, fidaxomicin, and metronidazole are the current standard of care to treat CDI; however, about 15 to 30% of the patients will develop recurrent episodes of CDI and, of these, up to 60% will experience additional relapses.^5^ Besides, *C. difficile* isolates with resistance or reduced susceptibility to the antibiotic have emerged globally.^6^ While microbial strategies to treat recurrent CDI through fecal microbiota transplantation (FMT) have seen great advancements, they are not Food and Drug Administration (FDA) approved and are associated with some risks, including limited knowledge of long-term health effects and the transfer of potentially fatal multidrug-resistant organisms to the patients, as was recently reported.^5,7^ And there is still no globally available vaccine for CDI. Thus, developing and testing alternative treatment modalities against CDI have become worldwide priorities because of the serious threat to public health.

Host immune responses to infection are increasingly being considered to play pivotal roles in CDI outcomes.^8^ Indeed, studies in mouse models, as well as human patients, have shown that the immune response to CDI can play both protective and pathogenic roles. For instance, MyD88 signaling, innate lymphoid cells (ILCs), leptin, IL-1β, IL-22, IL-25, IL-27, and IL-33, could provide protection against CDI.^9–16^ On the other hand, IL-17 and IL-23 could worsen *C. difficile*-associated severity and mortality.^17,18^ Together, these studies support the complexity of immune response during CDI and also suggest the potential of immunotherapeutic intervention to combat CDI. In fact, host-directed immunotherapy, which targets the host immune response to infection to either augment immunity or ameliorate immunopathology,^19^ is an emerging approach in the field of anti-infectives, as was recently demonstrated in COVID-19 and *Mycobacterium tuberculosis*.^20^ Understanding immune components that contributed essentially to the pathogenesis of CDI would have important implications for the development of host-directed therapies for CDI.

Progranulin (PGRN) is a key actor and regulator of multiple system functions in immune response and inflammation.^21^ There is emerging evidence that PGRN participates in the development of a variety of immune-mediated diseases, such as rheumatoid arthritis (RA), systemic lupus erythematosus (SLE), systemic sclerosis (SSc), and multiple sclerosis (MS).^22–26^ Recently, PGRN has been shown to play a central role in host defense against bacterial sepsis by promoting macrophage recruitment.^27^ However, in fungal sepsis, PGRN could aggravate lethal *Candida albicans* sepsis by regulating inflammatory response and antifungal immunity.^28^ Moreover, PGRN aggravated pulmonary immunopathology during influenza virus infection.^29^ Thus, PGRN-mediated immune responses can play both protective and pathogenic roles, which depend on the type of infectious agents. A previous study has demonstrated that PGRN protected against dextran sulfate sodium (DSS)- and picrylsulfonic acid (TNBS)-induced colitis progression in mice.^30^ This prompted us to study its role in the development of CDI. Here, we found that PGRN expression prevented CDI-associated mortality as a new guardian of the intestinal barrier. We saw that PGRN activated CD4^+^ T helper cells during CDI, increasing IL-22 production, improving intestinal barrier function, and promoting recovery from CDI-associated colitis. Therefore, PGRN is associated with host-protective immunity and gut barrier function and might be a promising host-targeted therapy in CDI.

## Materials and methods

### Ethics statement

This study was approved by the Clinical Research Ethics Committee of The First Affiliated Hospital of Chongqing Medical University (ethics review number 2020–341), and all subjects provided informed consent, and written informed consent was obtained from all subjects prior to participation according to the Declaration of Helsinki. All animal experiments were discussed with and approved by the Animal Care and Use Committee of the Chongqing Medical University and carried out according to the recommendations in the guide for the care and use of laboratory animals conformed to animal protection laws of China and applicable guidelines (YSDWBHF-R-27/08/2009).

### Human individulas

Patients with CDI were recruited at The First Affiliated Hospital of Chongqing Medical University (Chongqing, China). CDI positivity was assessed based on the presence of *C. difficile* toxins A/B in patient stool by VIDAS (bioMérieux, France), while CDI negativity was assessed based on the absence of *C. difficile* toxins A/B in patient stool by VIDAS.^14^ Human biopsies were obtained from the Department of Gastroenterology of The First Affiliated Hospital of Chongqing Medical University. CDI-negative samples were derived from patients suspected of various other intestinal diseases but confirmed negative for tissue pathology upon colonoscopy examination. Age- and sex-matched healthy control samples were obtained from healthy donors with no medical problems in the medical examination center of The First Affiliated Hospital of Chongqing Medical University. Ethical was approved by the Clinical Research Ethics Committee of The First Affiliated Hospital of Chongqing Medical University, and informed consent was obtained from all the participants. The characteristics of all the human subjects were included in supplementary Table S1.

### Mice

Progranulin-deficient mice (PGRN KO) were purchased from the Jackson Laboratory (Bar Harbor, ME, USA) and were backcrossed with a C57BL/6 background for at least 10 generations before use. C57BL/6-background IL-22 KO mice were purchased from the Shanghai Research Center for Model Organization (Shanghai, China). The wild-type (WT) C57BL/6 mice were obtained from Vital River Laboratories (Beijing, China). Both male and female mice (8-week-old) were age matched and evenly distributed within experimental groups, and sex- and age-matched controls were used in all experiments. All murine experiments were approved by the Institutional Animal Care and Use Committee of The First Affiliated Hospital of Chongqing Medical University (Permit Number: 2020–341). Mice were maintained on a 12 h-light/dark cycle and at a temperature of 20–26°C with 30–70% humidity in the specific pathogen-free animal facilities. To minimize suffering, all surgery was performed under pentobarbital sodium anesthesia (30 mg/kg, i.p), and every effort was made.

### Bacterial strain

*C. difficile* strain VPI 10,463 was obtained from the American Type Culture Collection (Manassas, VA). *C. difficile* was cultured and maintained at 37°C in brain – heart infusion (BHI) medium (BD Biosciences, Franklin Lakes, NJ) in an anaerobic environment (BBL GasPak Plus system; BD Biosciences).

### CDI murine model

Age- and sex-matched mice were provided with an antibiotic mixture of kanamycin (40 mg/kg/d), gentamicin (3.5 mg/kg/d), colistin (4.2 mg/kg/d), metronidazole (21.5 mg/kg/d), and vancomycin (4.5 mg/kg/d) in drinking water for 5 days, and then changed to regular water for two days. Subsequently, clindamycin (10 mg/kg) was injected intraperitoneally for 24 h, and after that, mice were challenged with 1 × 10^8^ or 1 × 10^9^ colony forming units (CFUs) of vegetative *C. difficile* (strain VPI 10,463) in 0.2 mL phosphate-buffered saline (PBS) via oral gavage.^31,32^ For vegetative cells infection, vegetative *C. difficile* was obtained by overnight culture of a plated single colony of *C. difficile* in anaerobic chopped meat broth (Anaerobic Systems), followed by a subculture of 100 μL in the same media for 5 h. Then 1 mL *C. difficile* in broth was pelleted, washed, quantified by spectrophotometer, resuspended to certain concentration in 0.2 mL PBS, and given orally by gavage. Quantification of *C. difficile* inoculum was verified by counting CFUs on anaerobic brain heart infusion (BHI) agar plates (Becton Dickinson) supplemented with taurocholate (Sigma) (BHI-T). As a control of an inactivated infective dose control, vegetative *C. difficile* was inactivated at 95°C for 30 min.^33^ The mice were monitored for daily weight and survival. All antibiotics were purchased from Sangon Biotech company.

### *C.* *difficile* quantification

Cecal contents were weighted and suspended in 75% alcohol, and the suspensions were diluted and cultured anaerobically on selective taurocholate-cycloserine-cefoxitin-fructose agar (TCCFA) plates at 37°C in an anaerobic chamber. Bacterial burden was determined by quantification of CFUs grown anaerobically on agar plates.

### Clinical scores

During the infection, mice were weighed and scored daily and euthanized if they developed severe disease (score >14) based on the scoring criteria. Clinical scores were calculated based on weight loss, coat appearance, eyes/nose discharge, activity, posture, and diarrhea as previously described.^14^

### Histopathology assessment

Mouse colon tissue sections were fixed in 10% formalin and transferred to 70% ethanol after 24 h. Sections were then paraffin-embedded, sliced, and stained with Hematoxylin and Eosin (H&E). Scoring was conducted by two independent, blinded observers. H&E tissue pathology scoring was conducted using numerical severity scores from 0 to 4 for each of the following parameters: epithelial disruption, submucosal edema, inflammatory infiltrate as previously described.^34^

### Immunofluorescence and immunochemistry

Colonic tissue slides were incubated with Anti-ZO1 tight junction (abcam, ab216880) or Occludin (E6B4R) Rabbit mAb (Cell Signaling Technology 91,131) at 4°C overnight, followed by incubation with a secondary antibody (Jackson ImmunoResearch, 711-165-152) for 60 min at room temperature. Then slides were incubated with DAPI (Sigma-Aldrich, MBD0015) for 5 min to stain the nuclei. For human biopsies, immunochemistry staining was performed using a primary antibody directed against PGRN (Novus, NBPI-32076) at a 1:100 dilutions and incubated at 37°C for 60 min. The tissue was stained using the Anti-Goat HRP-DAB Cell & Tissue Staining Kit (brown; R&D Systems, CTS008) and counterstained with hematoxylin (blue).

### TUNEL staining

Apoptosis in sections of colon sections was detected using a commercially available TUNEL staining kit (MedChemExpress, HY-K1078), according to the manufacturer’s instructions.

### Intestinal permeability assay

After fasted for 6 h, mice were orally administrated with 200 µL of 60 mg/mL fluorescein isothiocyanate (FITC)-dextran solution (average mol wt 4, 000; Sigma-Aldrich 469,944) for 4 h. The plasma was collected and diluted with same volume of PBS. FITC fluorescence (excitation 485 nm; emission 535 nm) was measured by microplate reader Fluoroskan (Thermo Fisher Scientific). The plasma FITC-dextran concentration was calculated from a standard curve generated by serial dilution of FITC-dextran in PBS.

### Tissue protein and cytokine analysis

Colon tissue (100 mg) was isolated and rinsed gently with ice-cold PBS, then homogenized in RIPA (Beyotime, P0013D) containing PMSF (Beyotime, ST507). The lysates were spun at 4°C, 12000 g for 5 min and the supernatants were transferred to a new tube for subsequent protein analysis. The protein levels of mouse progranulin (PGRN) were measured by Progranulin ELISA kits (R&D system, MPGRN0) according to the manufacturer’s instructions. Cytokines were determined by cytokine ELISA kits (Biolegend) for mouse TNF-α, IL-6, IL-1β, IL-22, IL-17A, IL-10, IL-4. Human serum PGRN and IL-22 were measured using the Human Progranulin DuoSet ELISA Kit (R&D system, DY2420) and ELISA MAX™ Deluxe Set Human IL-22 (Biolegend 434,504) according to the manufacturer’s instructions.

### Quantitative real-time PCR

Total RNA was extracted from colon tissues and cells using RNAiso Plus (TaKaRa, 9109). The cDNA was reversely transcribed from 1 µg total RNA of each sample using the PrimeScript RT reagent kit (TaKaRa, RR037A). The resulting cDNA was analyzed by real-time PCR using TB Green Premix Ex Taq II kit (TaKaRa, RR820A). Primers were synthesized by Sangon Biotech company, and the following primer pairs were used: GAPDH 5’-TGGCCTTCCGTGTTCCTAC-3’ forward and 5’-GAGTTGCTGTTGAAGTCGCA-3’ reverse; IL-22 5’- GTCAACCGCACCTTTATGCT −3’ forward and 5’- CCCCGATGAGCCGGACA −3’ reverse; RegIIIβ 5’- GAATATACCCTCCGCACGCA −3’ forward and 5’- GGTCATGGAGCCCAATCCAA −3’ reverse; RegIIIγ 5’- AGCCACAAGCAAGATCCCAA −3’ forward and 5’- GGCCATAGTGCACACAGAGT −3’ reverse V3-V4 region of the bacterial 16S rRNA 5’- ACTCCTACGGGAGGCAGCAG −3’ forward and 5’- GGACTACHVGGGTWTCTAAT −3’ reverse. The gene for glyceraldehyde-3-phosphate dehydrogenase (GAPDH) was amplified as an endogenous reference. Quantification was determined using comparative 2^−ΔΔ*Ct*^ methods.

### Western blot

Colon tissue was rinsed with ice-cold PBS and homogenated with liquid nitrogen and then was lysed with RIPA containing PMSF for 30 min on ice, followed by centrifuging at 4°C, 12000 g for 5 min. 5×loading buffer (Beyotime, P0286) was added to the supernatant, followed by boiling for 10 min. Each sample was loaded to 8–10% SDS-PAGE and blotted onto a polyvinylidene fluoride (PVDF) membrane (Millipore, ISEQ000010). After blocking with 5% skim milk, the membrane was incubated with primary antibodies overnight at 4°C. Next, the membrane was incubated with a secondary antibody Goat Anti-Rabbit IgG (Jackson ImmunoResearch, 111-035-003) for 1 h at room temperature. After incubating with the substrate, blots were detected by using the Bio-Rad Imager. Primary antibodies included: Anti-ZO1 tight junction (abcam, ab216880); Occludin (E6B4R) Rabbit mAb (Cell Signaling Technology 91,131); GAPDH (D16H11) XP Rabbit mAb (Cell Signaling Technology, 5174).

### Lamina propria cells isolation

For isolation of lamina propria cells, colons were cut longitudinally and rinsed thoroughly in iced-PBS containing 100 μg/mL penicillin and streptomycin (P/S) (Gibco 15,140,122). Colon tissues were laterally cut into pieces of 0.5–1.0 cm length and placed in PBS with 1 mM DTT (Beyotime, ST041) and 5 mM EDTA (Beyotime, ST069) at 37°C shaking incubator to remove intestinal epithelial cells. The colon tissue was cut into smaller pieces and transferred into 60 mm dishes then resuspend in 5 mL RPMI 1640 (Gibco, C11875500BT) supplemented with 10% Fetal Bovine Serum (Sigma-Aldrich, F8687), 1%P/S, 1 mg/mL collagenase IV (Sigma-Aldrich, C5138) and 150 μg/mL DNase I (Roche,10104159001) for 40 min at 37°C with 5% CO_2_. Cells were generated by passaging samples through 100 µm and 70 µm cell strainer (Biosharp, BS-100-XBS, and BS-70-XBS) and collected by centrifugation. Cells were further separated by 40% and 80% Percoll density gradient separation (GE Healthcare 17,089,102) to remove red cells and residual intestinal epithelial cells.

### Flow cytometry

Lamina propria cells were treated with a cell stimulation cocktail (plus protein transport inhibitors) containing phorbol 12-Myristate 13-Acetate (PMA), ionomycin, Brefeldin A, and Monensin (eBioscience, 00–4975) for 6 h, then washed once in 1 × DPBS (Gibco 14,190,144). For Live/Dead staining, cells were stained with Fixable Viability Stain 780 (BD Pharmingen 565,388). After Fc receptors blocking (CD16/32, BD Pharmingen 553,141), cells were stained with the following cell surface monoclonal antibodies: CD45 (BD Pharmingen 560,510), CD3e (BD Pharmingen 563,024), CD4 (BD Pharmingen 553,048), CD8a (BD Pharmingen 553,030), γδTCR (BD Pharmingen 562,892), NK1.1 (BD Pharmingen 550,627), Mouse Hematopoietic lineage antibody (eBioscience, 22-7770-72), CD127 (BD Pharmingen 121,112), then fixed and permeabilized using the Foxp3/Transcription Factor Fixation/Permeabilization set (eBioscience, 00-5523-00) in the dark for 30 min. Intracellular and intranuclear cytokines were then stained with antibodies: IL-22 monoclonal antibodies (eBioscience, 46-7221-82) and anti-RORγt (BD Pharmingen 562,894). Flow cytometry data was acquired by BD FACS Diva software and analyzed using Flowjo (Version 10.8.1).

### CD4^+^ T cells isolation and treatment

Mouse splenic CD4^+^ T cells were purified from murine spleen by EasySep immunomagnetic negative selection (StemCell Technologies 19,852). CD4^+^ T cells were seeded in the 48-well plates (Corning, 3548) and activated with 5 µg/mL anti-CD3 mAb (Bio X Cell, BE0001–1) and 2 µg/mL anti-CD28 mAb (Bio X Cell, BE0015–1) in the presence or absence of recombinant mouse PGRN (R&D System, 2557-PG-050), under neutral (without exogenous cytokines) conditions. Cells were cultured for 48 h at 37°C with 5% CO_2_. Human peripheral blood CD4^+^ T cells were isolated from the peripheral blood of healthy volunteers using human CD4^+^ T cell isolation kit (Miltenyi Biotec, 130-096-533). Cells were activated with 5 µg/mL anti-human CD3 (Bio X Cell, BE0001–2) and 2 µg/mL anti-human CD28 (Bio X Cell, BE0248) in the presence or absence of recombinant human PGRN (R&D system, 2420-PG-050) for 48 h in 37°C chamber.

For CD4^+^ T cell staining, cells were treated with Brefeldin A (Biolegend 420,601) to employ protein transporter blockage for 6 h, then harvested and stained with cell surface marker antibodies: anti-mouse CD3 (BD Pharmingen 553,061) and anti-mouse CD4 (BD Pharmingen 553,048) or anti-human CD3 (BD Pharmingen 561,806) and anti-human CD4 (BD Pharmingen 562,970). Next, the cells were fixed and permeabilized using Fixation/Permeabilization kit (BD Pharmingen 554,714) at 4°C for 30 min in the dark. Intracellular cytokine was stained with anti-mouse IL-22 (eBioscience, 46-7221-82) or anti-human IL-22 (BD Pharmingen 567,161). All events were acquired by Beckman Coulter Cytoflex software, and data analysis was performed via Flowjo (Version 10.8.1).

### Cell depletion and antibody neutralization

For CD4 T cell depletion, WT mice were administrated with 400 μg anti-mouse CD4 mAb (BioXcell, BE0003–1) or IgG isotype control (BioXcell, BE0090) on days −6, −3 and on the day of infection with *C. difficile*.^35^ For PGRN neutralization, WT mice were administrated with 100 μg anti-PGRN (R&D Systems, AF2557) or IgG isotype control (R&D Systems, MAB006) on days −1, 1 and 2 of *C. difficile* infection.

### DNA extraction and 16S rRNA sequencing and analysis

Total microbial genomic DNA was extracted from mice cecal contents using the PF Mag-Bind Stool DNA Kit (Omega) according to manufacturer’s instructions. The hypervariable region V3-V4 of the bacterial 16S rRNA gene was amplified by an ABI GeneAmp® 9700 PCR thermocycler. Purified amplicons were sequenced on an Illumina PE300 platform (Illumina) according to the standard protocols by Majorbio Bio-Pharm Technology Company (Shanghai, China). The raw sequencing reads were deposited into the NCBI database under the accession number PRJNA1121310. The data were analyzed on the free online platform of Majorbio Cloud Platform.

### Statistical analysis

For murine work, survival curves were estimated using a Log-Rank (Mantel-Cox) statistical test. Comparisons between two groups were conducted using a student’s t-test or a nonparametric Mann – Whitney U test depending on whether the data were normally distributed. Comparisons among three or more groups were performed with one-way ANOVA. ELISA data from patients were analyzed to assess the correlation between PGRN and IL-22 expression using Spearman correlation. Statistical analyses were performed with Graphpad Prism 8.0 software. Values of *p* < 0.05 were considered statistically significant.

## Results

### PGRN provided protection to mice against CDI-associated mortality and morbidity

**Figure 1.**
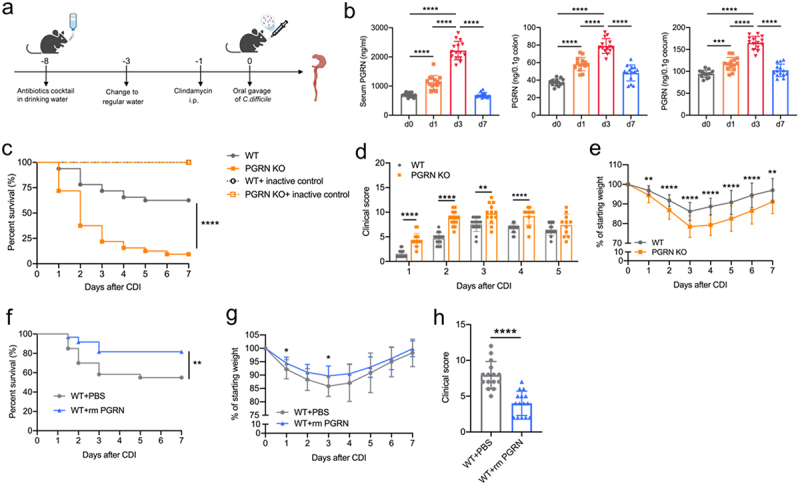
PGRN provided protection against cdi-associated mortality and morbidity. (a) Visual abstract of CDI model used in this study. (b) WT mice were infected with 1 × 10^9^ colony forming units (CFUs) of *C. difficile* (VPI 10,463) after antibiotics cocktail pretreatment for 5 days, and then PGRN protein levels in the blood, colon and cecum were measured by ELISA at the indicated time points after CDI (*n* = 15). (c) WT and PGRN KO mice were gavaged with 1 × 10^9^ CFUs *C. difficile* (or heat-inactived control). After CDI, the survival curves of four groups of mice (*n* = 32). (d-e)WT and PGRN KO mice were gavaged with 1 × 10^9^ CFUs *C. difficile*. (d) clinical scores (*n* = 11-15) and (e) weight loss (*n* = 20-33) were recorded daily. (f-h) WT mice were orally infected with 1 × 10^9^ CFUs *C. difficile* and treated with vehicle control (PBS) or recombinant mouse PGRN (rmPGRN,10 μg/mouse). (f) Survival curves (*n* = 30), (g) weight loss (*n* = 15) and (h) clinical scores on day 2 (*n* = 15). Survival curves were compared using a log rank statistical test (c and f). Data were expressed as mean ± SD. Statistical significance was tested by one-way ANOVA for multiple comparisons (b, d, e, g), or two-tailed paired Student t-test (h). *p < 0.05, **p < 0.01, ***p < 0.001, ****p < 0.0001.

To assess the relevance of PGRN during CDI, we first quantified PGRN protein in WT mice challenged with *C. difficile* by oral gavage (Figure 1a). Compared with uninfected controls, CDI resulted in a significant increase in PGRN expression in the blood, colon, and cecum (Figure 1b). Antibiotic treatment had no effects on the production of PGRN in the colon and cecum of WT mice (supplementary Figure S1), suggesting that the production of PGRN is independent on the microbiota.

To evaluate the role of PGRN in murine CDI, we infected antibiotic-treated WT and PGRN knockout (KO) mice with *C. difficile*. Because microbiota plays an important role in the pathogenesis of CDI, we first assessed whether PGRN loss altered the microbiota composition by 16S ribosomal RNA analysis from cecal contents. We found no significant differences in microbiota diversity in antibiotic-treated (ABX) WT and PGRN KO mice prior to CDI (supplementary Figure S2a-d). In a mild CDI model established with a dose of 1 × 10^8^ CFUs of VPI 10,463 as described previously,^13,31^ the survival rate of PGRN KO mice was lower than that of WT mice, but it did not reach statistical significance (Supplementary Figure 3). In a severe CDI model established with a dose of 1 × 10^9^ CFUs of VPI 10,463, the survival rate of PGRN KO mice was significantly lower than that of WT mice (Figure 1c). Therefore, our subsequent experiments were performed in a severe *C. difficile* infection model with 1 × 10^9^ CFUs of VPI 10,463 challenge. PGRN KO mice had significantly more severe clinical scores based on parameters including weight loss, piloerection, ocular discharge, activity, posture, and diarrhea (Figure 1d), and displayed dramatically increased weight loss during severe CDI (Figure 1e). To rule out developmental abnormalities in PGRN KO mice as a confounding factor, we used a second murine model, in which PGRN was blocked using an antibody directed toward PGRN.^27^ Mice treated with anti-PGRN antibody had a significantly increased mortality when compared with isotype control mice (supplementary Figure S4a). Concurrently, PGRN – blocked mice had significantly increased clinical scores as compared with isotype controls (supplementary Figure S4b).

To further determine if PGRN mediates protection during CDI, we tested if supplementation of recombinant PGRN protein could reduce disease severity in the setting of antibiotics and active CDI. Our PGRN treatment regimen caused a significant increase in PGRN protein levels within the colon prior to infection (supplementary Figure S5a), and this PGRN treatment did not significantly alter the microbiota composition prior to CDI (supplementary Figure S5b-e). Notably, PGRN treatment dramatically increased survival in WT mice following CDI (Figure 1f). Consistently, PGRN-treated mice had significantly reduced weight loss (Figure 1g), and less severe clinical scores (Figure 1h). Besides, PGRN treatement also protected PGRN KO mice against death after CDI (supplementary Figure S6). Taken together, these results above suggested a protective role for PGRN in murine CDI.

### Absence of PGRN aggravated CDI-induced intestinal inflammation and barrier dysfunction in mice

**Figure 2.**
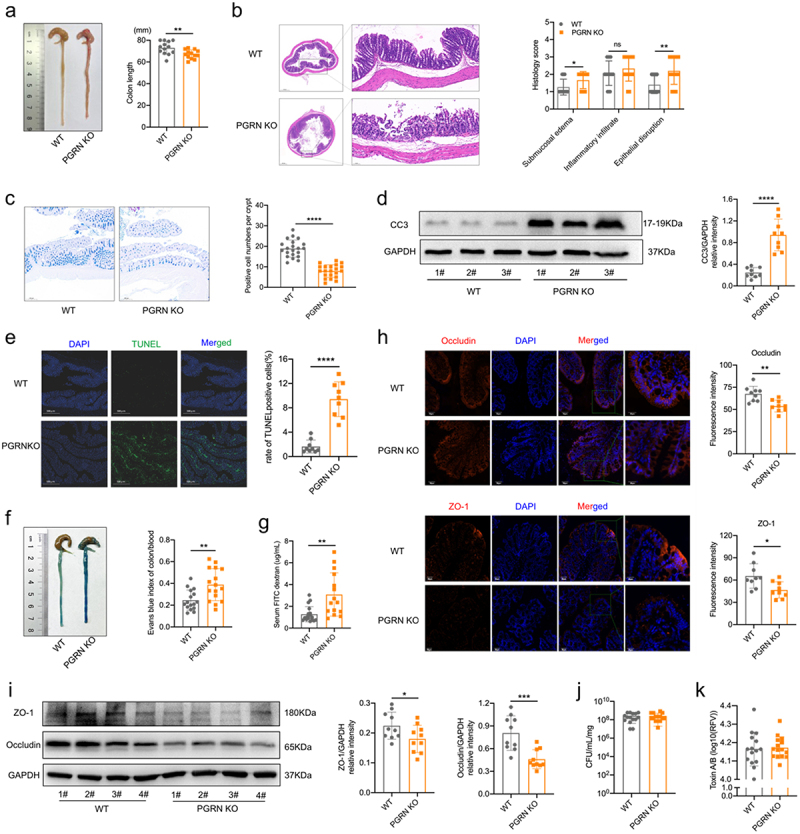
Absence of PGRN aggravated cdi-induced intestinal inflammation and barrier dysfunction in mice. WT and PGRN KO mice were gavaged with *C. difficile* (1 × 10^9^ CFUs) prior to antibiotics cocktail and sacrificed on day 2. (a) Representative image (left) and colonic length (right) on day 2 (*n* = 13). (b) Representative images of hematoxylin and eosin-stained colonic sections (left) and histological scores (right) in mice (*n* = 15). (c) Representative images of Alcian blue and periodic acid-Schiff (AB-PAS)-stained colonic sections and the number of goblet cells was quantified (*n* = 7). (d) Representative immunoblot images (left) and quantification analysis (right) of cleaved caspase 3 (CC3) protein expression of colonic tissues (*n* = 9), GAPDH was used as a loading control. (e) Representative images (left) and rate of positive cells (right) of TUNEL (FITC) stained colonic sections on day 2 (*n* = 9). (f) Representative colon Evans blue staining (left) and Evans blue index (right) of indicated mice are shown (*n* = 16). (g) FITC dextran detection assay in the serum was used to determine gut permeability (*n* = 15-18). The expression levels of tight junction proteins occludin and ZO-1 were observed by (h) immunofluorescence (left) and fluorescence intensity (right) in mice (*n* = 9) and (i) Western blot (representative immunoblot images, left) and quantification analysis (right) in mice (*n* = 10), GAPDH was used as a loading control. (j) *C. difficile* bacterial burden in cecal contents (*n* = 15). (k) Toxin A/B in cecal contents were assessed by VIDAS (*n* = 15). Scale bar is 50 μm and 25 μm. Data were expressed as mean ± SD. Statistical significance was tested by two-tailed unpaired Student t-test. *p < 0.05, **p < 0.01, ***p < 0.001, ****p < 0.0001.

Exacerbated mortality of PGRN KO mice was associated with significantly increased colon shortening than WT mice during CDI (Figure 2a). Histological evaluation of the colon by H&E staining showed that PGRN loss resulted in a significant increase in epithelial damage and sub-mucosal edema (Figure 2b), and the number of goblet cells per upper crypt in the colonic mucosa was significantly decreased in PGRN KO mice when compared with WT mice during CDI (Figure 2c). Immune cell apoptosis in the intestine is also accompanied by CDI.^36^ As predicted, cleaved caspase 3 (CC3) expression, a marker for apoptosis, was significantly enhanced in the colons from PGRN KO mice when compared with WT mice during CDI (Figure 2d). DNA fragmentation analysis by terminal deoxynucleotidyl transferase dUTP nick end labeling (TUNEL) confirmed that PGRN loss significantly increased cell apoptosis in the colon during CDI (Figure 2e). Accordingly, PGRN KO mice had significantly increased vascular permeability to Evans blue dye in the colon (Figure 2f), as well as gut permeability as quantified by FITC-dextran gut-barrier permeability assay (Figure 2g). Furthermore, dramatically decreased expression levels of tight junction proteins relating to intestinal barrier function including Occludin and zonula occludens-1 (ZO-1) were observed in the colon tissues from PGRN KO mice when compared with WT mice in response to CDI (Figure 2h, i).

Despite the increase in epithelial damage post CDI, PGRN KO mice had similar levels of *C. difficile* bacterial burdens (Figure 2j) and virulence factors, toxins A and B (Figure 2k), in the cecal contents. Together, these results indicate that PGRN loss may aggravate disease severity and mortality during CDI by aggravating intestinal inflammation and barrier dysfunction, rather than by augmenting *C. difficile* growth or toxin production.

### Absence of PGRN resulted in decreased IL-22 production during murine CDI

**Figure 3.**
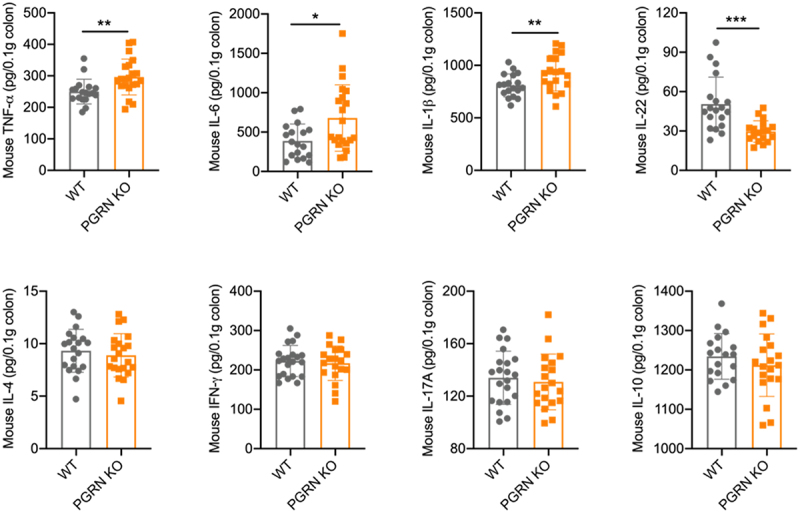
PGRN regulated cytokine production during murine CDI. WT and PGRN KO mice were orally infected by *C. difficile* (1 × 10^9^ CFUs) after antibiotics cocktail for 5 days. Cytokines of colonic tissues were quantified by ELISA after infection on day 2 (*n* = 18-21). Data were expressed as mean ± SD. Statistical significance was tested by two-tailed unpaired Student t-test. *p < 0.05, **p < 0.01, ***p < 0.001.

The interplay between immune responses and the intestinal epithelia is critical to maintain balance of inflammatory response and homeostasis of gut barrier, and previous studies have shown that the type of host immune response can determine the severity and outcome of infection in CDI.^15,17^ To study if PGRN shaped host immunity during CDI, we evaluated the cytokine milieu in the colon tissue. In consistent with increased mortality, PGRN KO mice had significantly higher protein levels of inflammatory cytokines including TNF-α, IL-6, and IL-1β than WT mice post CDI (Figure 3). However, there was no significant difference in the protein levels of IFN-γ, IL-4, IL-17A, and IL-10 between PGRN KO and WT mice. Interestingly, IL-22 protein levels in the colon tissues from PGRN KO mice were significantly lower than those in WT mice during CDI, which was validated by quantitative real-time PCR analysis showing that IL-22 mRNA levels in colon tissues were also significantly decreased in PGRN KO mice post CDI (supplementary Figure S7a). Besides, the expression levels of IL-22-associated downstream target genes, RegIIIβ and RegIIIγ, were also significantly down-regulated in PGRN KO mice when compared with WT mice post CDI (supplementary Figure S7b). Since IL-22 is known to have a protective role in CDI,^13^ we concluded that the reduction in IL-22 may contribute to the increased mortality observed in PGRN KO mice during CDI.

### PGRN was required for IL-22 production by CD4^+^ T helper cells during CDI

**Figure 4.**
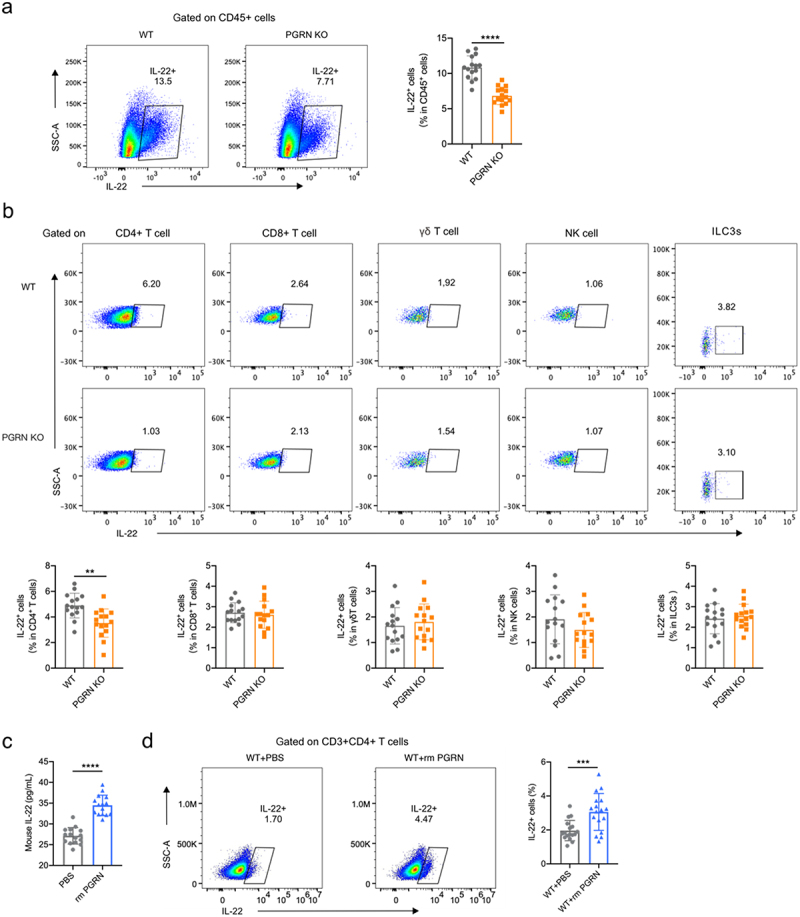
PGRN was required for IL-22 production by CD4^+^ T helper cells during CDI. (a-b) WT and PGRN KO mice were orally administrated with *C. difficile* (1 × 10^9^ CFUs) after antibiotics cocktail. (a) Production of IL-22 in colonic CD45^+^ cells (pan-leucocytes) were measured by flow cytometry on day 2 (*n* = 15). (b) The frequency and production of IL-22 in colonic CD4^+^ T helper cells, CD8^+^ T helper cells, γδ T cells, NK cells and ILC3 were detected by FACS method on day 2 (*n* = 15). (c) Splenic CD4^+^ T cells from uninfected WT mice were treated with or without rm PGRN (1 μg/mL). IL-22 production in supernatant was measured at 48 hours by ELISA (*n* = 15). (d) Splenic CD4^+^ T cells from uninfected WT mice were actived by anti-mouse CD3/CD28 mAbs and treated with or without rm PGRN (1 μg/mL). IL-22 production in CD4^+^ T helper cells were measured by flow cytometry on day 2 (*n* = 17). Data were expressed as mean ± SD. Statistical significance was tested by two-tailed unpaired Student t-test. **p < 0.01, ***p < 0.001, ****p < 0.0001.

αβ T cells, natural killer (NK) cells, γδ T cells, and innate lymphoid cells (ILCs) are producers of IL-22.^37^ To determine the phenotype of IL-22-producing cells regulated by PGRN during CDI, intestinal cells in the colon tissues were isolated after CDI, stained for cell markers and cytoplasmic IL-22, and analyzed by flow cytometry. PGRN deficiency did not affect the frequency of immune cells in the colonic lamina propria, including CD45^+^ cells (pan-leucocytes) and ILC3 cells (supplementary Figure 8a, b). In the absence of any *ex vivo* stimulation, we found that the frequency and production of IL-22 in CD45^+^ cells recovered from PGRN KO mice were significantly lower than those in WT mice post CDI by intracellular staining (supplementary Figure 8c and Figure 4a). Further analysis of the cellular source of IL-22 revealed that there was a significant decrease in IL-22-producing CD4^+^ T helper cells in the colons from PGRN KO mice compared with WT mice after CDI (supplementary Figure 9a and Figure 4b). However, the frequency and production of IL-22 in CD8^+^ T helper cells, γδ T cells, NK cells, or ILC3 were not affected by PGRN loss during CDI. To assess whether PGRN can directly induce IL-22 production by CD4^+^ T helper cells, CD4^+^ T helper cells from the spleens of uninfected WT mice were assessed for IL-22 production. PGRN *ex vivo* stimulation was associated with a significant induction of IL-22-producing CD4^+^ T helper cells (supplementary Figure 9b and Figure 4c, d).

### Restoration of IL-22 provided protection to PGRN KO mice against CDI-associated mortality and morbidity

**Figure 5.**
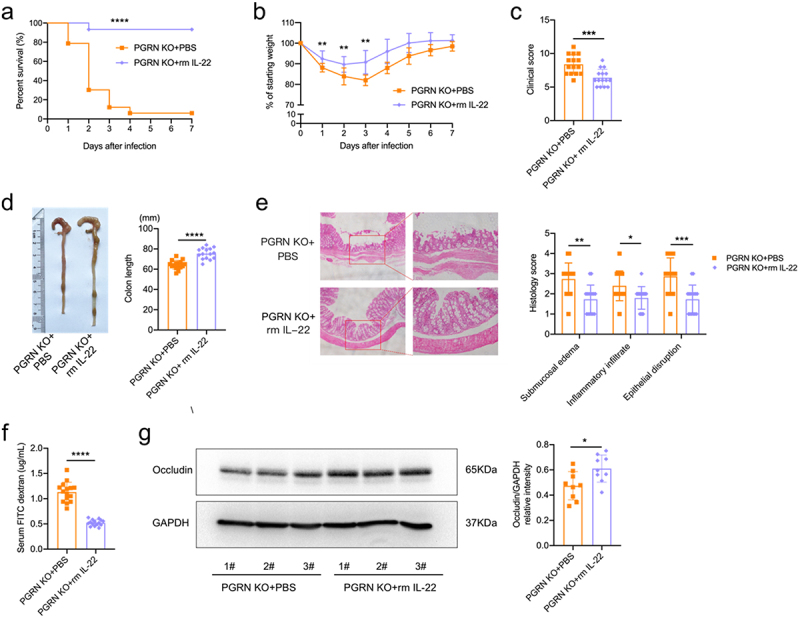
Restoration of IL-22 provided protection against CDI in PGRN KO mice. PGRN KO mice were gavaged with *C. difficile* (1 × 10^9^ CFUs) and treated with recombinant mouse IL-22 (rmIL-22, 1 μg/mouse) or vehicle control (PBS) by intraperitoneal injection. Mice were weighed daily. (a) Survival curves (*n* = 30-33). (b) Weight loss (*n* = 14). (c) Clinical scores on day 2 (*n* = 15). (d) Representative image and colonic length in PGRN KO mice with or without rm IL-22 on day 2 (*n* = 15). (e) Representative images of colon hematoxylin and eosin-stained staining (left) and histological score (right) on day 2 (*n* = 15). (f) Serum levels of FITC-dextran were measured (*n* = 15). (g) Representative immunoblot images (left) and quantification analysis (right) of Occludin protein expression of colonic tissues (*n* = 9), GAPDH was used as a loading control. Survival curves were compared using a log rank statistical test (a). Data were expressed as mean ± SD. Statistical significance was tested by one-way ANOVA for multiple comparisons (b), or two-tailed paired Student t-test (c-f). *p < 0.05, **p < 0.01, ***p< 0.001, ****p< 0.0001.

Having observed decreased IL-22 production in PGRN KO mice during CDI, we tested if repletion of IL-22 could reduce disease severity and mortality in PGRN KO mice upon CDI. Our IL-22 treatment regimen caused a significant increase in IL-22 protein levels within the colon prior to infection (supplementary Figure 10a). IL-22 treatment significantly increased survival in PGRN KO mice following CDI (Figure 5a). In addition, IL-22-treated PGRN KO mice had reduced weight loss (Figure 5b), and less severe clinical scores during CDI (Figure 5c). PGRN KO mice treated with IL-22 had significantly longer colon length (Figure 5d). Histological evaluation of the colon tissues from PGRN KO mice showed that IL-22 significantly decreased CDI-associated tissue pathology (Figure 5e), and IL-22 significantly reduced IL-1β and IL-6 levels in PGRN KO mice post CDI (supplementary Figure 10b). Accordingly, IL-22-treated PGRN KO mice had reduced gut permeability as quantified by FITC-dextran gut-barrier permeability assay (Figure 5f), and increased protein levels of Occludin were observed in the colon tissues from PGRN KO mice treated with IL-22 when compared with PBS-treated PGRN KO mice post CDI (Figure 5g). We also noticed that IL-22- and PBS-treated PGRN KO mice had similar levels of *C. difficile* bacterial burdens and toxins A/B (supplementary Figure 11a, b).

### PGRN protected against CDI-associated mortality and morbidity depending on CD4^+^ T cells and IL-22

**Figure 6.**
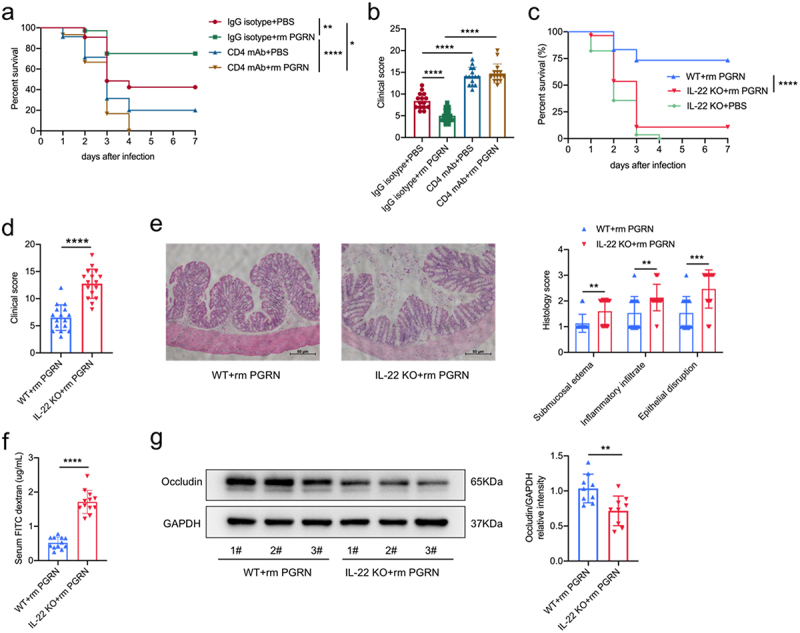
PGRN protected against cdi-associated mortality and morbidity depending on CD4^+^ T helper cells and IL-22. (a-b) WT mice were orally infected with *C. difficile* (1 × 10^9^ CFUs) and treated with or without recombinant mouse PGRN (rmPGRN, 10 μg/mouse). Mice were administrated with isotype antibody (25 mg/kg), or anti-CD4 antibody (25 mg/kg) intraperitoneally on days − 6, −3 and on the day of infection with *C. difficile*. (a) Survival curves (*n* = 30-35). (b) Clinical scores were assessed on day 2 (*n* = 15). (c-g) WT and IL-22 KO mice were treated with antibiotic cocktail prior to infection with *C. difficile* and treated with rm PGRN (10 μg/mouse) or vehicle control (PBS) intraperitoneally. (c) Survival curves (*n* = 28-30). (d) Clinical scores on day 2 after CDI (*n* = 16). (e) Colonic histopathology was assessed on day 2 after CDI (*n* = 15). (f) Content of FITC-dextran in serum on day 2 after CDI (*n* = 12). (g) Representative immunoblot images (left) and quantification analysis (right) of Occludin protein expression of colonic tissues (*n* = 9) on day 2 after CDI, GAPDH was used as a loading control. Scale bar is 50 µm. Survival curves were compared using a log rank statistical test (a and c). Data were expressed as mean ± SD. Statistical significance was tested by one-way ANOVA for multiple comparisons (b), or two-tailed paired Student t-test (d-f). *p < 0.05, **p < 0.01, ***p< 0.001, ****p < 0.0001.

To further establish the role of PGRN-mediated IL-22 production from CD4^+^ T helper cells in CDI, we firstly treated mice by intraperitoneal injection of anti-CD4 monoclonal antibody. Depleting CD4^+^ T helper cells by anti-CD4 treatment completely abrogated the protective effects of PGRN on CDI-associated mortality and morbidity (Figure 6a, b) and also increased the mortality and morbidity of untreated mice after CDI, demonstrating that CD4^+^ T helper cells were an essential downstream effector cell in PGRN-mediated protection. Furthermore, PGRN-mediated protection from mortality and morbidity after CDI was lost in IL-22 KO mice (Figure 6c, d). In line with these findings, PGRN-treated mice lacking IL-22 showed increased CDI-associated tissue pathology (Figure 6e), and PGRN-treated IL-22 KO mice had increased gut permeability and reduced protein expression of Occludin in the colon tissues compared with PGRN-treated WT mice (Figure 6f, g). Besides, we found comparable levels of *C. difficile* bacterial burdens and toxins A/B in IL-22 KO and WT mice treated with PGRN (supplementary Figure 12a, b). Together, these data suggest that CD4^+^ T helper cells were implicated in PGRN-mediated protection from CDI via IL-22 production.

### PGRN induced IL-22 expression in human CD4^+^ T helper cells

**Figure 7.**
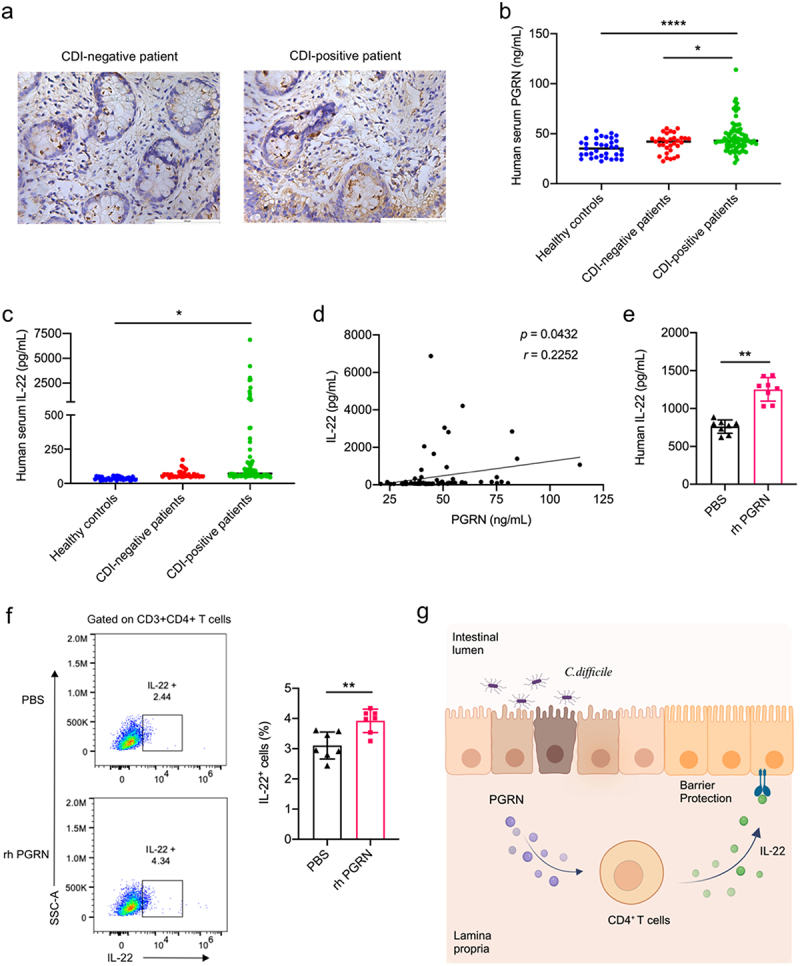
PGRN induced IL-22 expression in human CD4^+^ T helper cells. (a) Immunohistochemical staining for PGRN in a representative colon biopsy specimen from a CDI-negative patient (left) and a CDI-positive patient (right). (b-c) serum samples from 80 *C. difficile*-positive patients, 32 *C.Difficile*-negative patients with diarrhea, and 35 healthy individuals were assayed for PGRN and IL-22 by ELISA. (d) Spearman correlation between PGRN and IL-22 concentrations. (e) Peripheral blood CD4^+^ T cells isolated from healthy individuals were treated with or without recombinant human PGRN (rh PGRN, 1 μg/mL) for 2 days. IL-22 production was analyzed by ELISA (*n* = 8 biologically independent samples). (f) Peripheral blood CD4^+^ T cells isolated from healthy individuals were activated with anti-human CD3/CD28 mAbs in the presence or absence of recombinant human PGRN protein (1 μg/mL). IL-22 production was analyzed by flow cytometry (*n* = 7 biologically independent samples). (g) Schematic representation of the mechanism by which PGRN protects CDI via IL-22 induction of CD4^+^ T helper cells. Scale bar is 100 µm. Horizontal bars represent median values, and dots represent individual participants. Data were expressed as mean ± SD. Statistical significance was tested by one-way ANOVA for multiple comparisons (b and c), or two-tailed paired Student t-test (e and f). *p< 0.05, **p< 0.01, ****p < 0.0001.

To ascertain the relevance of PGRN in human CDI, we stained biopsy specimens of CDI-negative and CDI-positive patients. CDI-negative tissues were taken from patients who had suspected colitis but who had confirmed normal pathologic findings upon biopsy. Notably, significant inductions in PGRN expression were observed in CDI patients when compared to negative controls (Figure 7a). In a cohort of 80 human CDI patients (Supplementary Table S1), we detected significantly higher serum levels of PGRN in CDI-positive patients when compared with CDI-negative patients and healthy controls (Figure 7b). The levels of IL-22 in the sera of patients with CDI were also significantly increased compared with healthy controls, but they were not significantly altered compared with and CDI-negative patients (Figure 7c). Besides, there was a significant and positive correlation between PGRN and IL-22 in the sera of human CDI patients (Figure 7d). In addition, treatment with human PGRN could activate human CD4^+^ T cells to augment IL-22 production at protein level (supplementary Figure 13 and Figure 7e, f).

## Discussion

Our study illustrates a previously unrecognized role for PGRN in the protection from CDI-associated mortality and morbidity via – at least in part – the action of IL-22 in CD4^+^ T helper cells. We see that PGRN, a growth factor which was not regulated by the microbiota, was increased in the humans and mice with CDI. Loss of PGRN aggravated disease severity, despite the presence of equivalent levels of *C. difficile* and toxins in the cecal contents. PGRN loss resulted in increased mortality and morbidity and exacerbated intestinal barrier disruption during CDI. We further demonstrated that PGRN treatment protected against CDI in a CD4^+^ T cell- and IL-22-dependent manner. To our knowledge, our report is the first demonstrating that PGRN activates CD4^+^ T helper cells in the large intestine and induces IL-22-associated repair pathways that protect from CDI-mediated epithelial barrier damage, which may serve as a therapeutically important pathway that maintains integrity of the intestinal barrier and reduces mortality during CDI (Figure 7g).

Our previous results have shown that PGRN production was elevated during systemic and pulmonary infections caused by Gram-negative and Gram-positive bacteria, which contributed importantly to host defense by increasing bacterial clearance.^27,38^ However, this study adds another layer to our understanding of PGRN’s relevance to gut bacterial immunity. Our work demonstrated that PGRN was elevated upon mouse *C. difficile* bacterial infection, and its homeostatic expression was not regulated by the microbiota. PGRN-deficient mice were highly susceptible to *C. difficile* using a severe CDI model established with a high dose of 1 × 10^9^ CFUs of *C. difficile* strain VPI 10,463, whereas others have used a mild CDI model established with a dose of 1 × 10^8^ CFUs of VPI 10,463.^31^ Furthermore, PGRN supplementation dramatically enhanced the survival of mice in response to severe CDI. Although PGRN protected the host from bacterial pneumonia by enhancing the elimination of *P. aeruginosa* or *S. aureus*,^38^ PGRN did not affect the number of *C. difficile* or the level of toxin in the cecal contents, indicating that PGRN might promote host survival through mechanisms distinct from pathogen elimination or intoxication during CDI. The most profound impact was the worsened destruction of intestinal barrier function in PGRN-deficient mice upon CDI. We found that PGRN loss resulted in decreased protein expression levels of Occludin and ZO-1 in CDI, which are essential for maintaining epithelial junctional integrity.^37^ Therefore, PGRN plays a protective role in CDI by regulating intestinal barrier function and maintaining intestinal homeostasis, rather than by dampening *C. difficile* proliferation or toxin release in the cecal contents, which is similar with the beneficial roles of IL-25 or IL-33 in CDI.^14,16^

The induction of IL-22 by PGRN appears to be a major factor in enabling protection against CDI in mice. IL-22 produced by both innate and adaptive lymphocytes is indispensable for maintaining colonic epithelial barrier by preventing cell death, stimulating proliferation of epithelial stem cells, and inducing protective factors such as mucins and antimicrobial peptides from goblet and Paneth cells, respectively.^37,39,40^ IL-22-deficient mice had increased morbidity and mortality after CDI despite a comparable degree of intestinal pathogen burden,^41^ as we have observed in PGRN-deficient mice in this study. Since IL-22 did not influence the burden of *C. difficile* in the intestine, it is likely that its action occurred downstream and involved maintaining the intestinal barrier. PGRN supplementation did not protect IL-22-deficient mice from CDI, while restoration of IL-22 provided protection to PGRN-deficient mice against CDI-associated mortality and morbidity. Here, we provided evidence that PGRN is a novel inducer of IL-22 and is involved in gut immunity of CDI via the induction of IL-22. Thus, PGRN-mediated IL-22 elevation may protect against CDI by inducing tissue remodeling and repair pathways to strengthen the intestinal barrier function.

IL-22 can arise from several cell types in the intestine, including CD4^+^ T helper cells (Th22 cells), ILCs, NK cells, and γδ T cells.^41^ Among these, ILCs predominantly produce IL-22 without the requirement of antigen specificity and subsequent clonal expansion during the early stages of infection, whereas CD4^+^ T cells become the main source of IL-22 during the later stages of infection.^41,42^ A previous study has shown that *Clostridia* colonization of antibiotic-treated neonatal mice induces IL-22 production by ILCs and CD4^+^ T cells.^43^ In this study, we investigated the cellular source of intestinal IL-22 regulated by PGRN during CDI and found that CD4^+^ T cells accounted for the decreased IL-22 production almost the entire IL-22-staining cell population in the colon tissues from PGRN-deficient mice upon CDI. CD4^+^ T cells were identified as the effector cells by which PGRN protected against CDI-associated mortality. This result is at odds with a report by Abt MC et al., which showed that there were no significant differences in CDI outcomes between WT and Rag1 KO mice.^10^ The discrepancy between our data and the dataset of Abt MC et al. is likely due to the severity of CDI.^10^ We used a severe CDI model established with a high dose of 1 × 10^9^ *C. difficile* (VPI 10,463), whereas Abt MC et al. used a mild CDI model established with a dose of 200 spores of C. *difficile* (VPI 10,463 strain).^10^ Another variable that influences the mortality rate is the method, which was different in the two studies: we used CD4 monoclonal antibody to deplete CD4^+^ T helper cells, while Abt MC et al. used Rag1 KO mice that lack mature T and B lymphocytes.^10^ Although ILC3s have been reported as a major source of IL-22 during CDI,^10,44^ we found no significant differences between PGRN-deficient mice and WT mice in the percentage of IL-22–producing ILC3s during CDI, suggesting that PGRN does not regulate the induction of IL-22 by ILC3s upon CDI. Thus, IL-22 induction in ILC3s of innate immune system may rapidly respond to CDI and provide initial host defenses in the intestine, while IL-22 production in CD4^+^ T helper cells of the adaptive immune system elicited by PGRN are also of high importance during CDI by reducing the severity of disease. In contrast to its inhibition effects on Th1/Th17 cells in experimental autoimmune uveitis and experimental autoimmune encephalomyelitis,^45^ PGRN acts as an upstream regulator of CD4^+^ T helper cell function and barrier protection during CDI by integrating signals from IL-22 production to promote repair. However, the cellular sources of PGRN in the gut during CDI remain unknown, and it remains to be determined whether *C. difficile* regulated PGRN expression directly or indirectly. Furthermore, the mechanisms by which PGRN modulates T cell activation but not ILCs in CDI remain unknown. Finally, the severity of CDI depends on the *C. difficile* challenge dose.^46^ Here in a severe CDI model, about 70% WT mice survived upon the infection of 1 × 10^9^ CFUs of VPI 10,463, while in a mild CDI model established with a dose of 1 × 10^8^ CFUs of VPI 10,463, about 100% WT mice survived.^31^ Besides, *C. difficile* ATCC BAA1870 at 1 × 10^10^ CFUs/mL in 0.5 mL normal saline has been used to establish *C. difficile*-associated diarrhea (CDAD) mouse model.^32^ How can we improve animal models so that they more closely resemble CDI in humans? Future studies are required to answer these questions above.

The effect of PGRN on mouse CD4^+^ T helper cells can be extended to human CD4^+^ T helper cells. Compared with healthy controls, the levels of PGRN in the sera of patients with CDI were markedly increased, which positively correlated with the levels of IL-22. These incseased PGRN levels may be a reactive process to CDI, which do not achieve full protection. Similar observations have been made for IL-27 and IL-33,^15,16^ which would share with PGRN protective actions upon CDI. In conclusion, this study has revealed that PGRN induces an epithelial barrier repairing-related cytokine, IL-22, in CD4^+^ T helper cells. Our study provides the first evidence for a functional linkage between these two host factors, strengthening the role of activating PGRN-IL-22 axis in reestablishing the intestinal epithelial barrier during CDI. Considering that persistent diarrhea in CDI correlated with intestinal inflammation and not fecal pathogen burden in adults and children with CDI,^47,48^ PGRN administration might represent a novel host-directed therapy in this enteric infection, as an alternative approach to the pathogen-targeted therapies.

## Supplementary Material

3rd Supplementary Figures and Table.docx

## Data Availability

All data needed to evaluate the conclusions in the paper are present in the paper and/or the Supplementary Materials. Additional data related to this paper may be requested from the authors.
