# Supplementary material for: Progranulin protects against Clostridioides difficile infection by enhancing IL-22 production
Source: Gut Microbes. 2024 Sep 30;16(1):2409220. doi: 10.1080/19490976.2024.2409220 (PMC11444516; doi:10.1080/19490976.2024.2409220)
Supplement: 3rd Supplementary Figures and Table.docx [file KGMI_A_2409220_SM4405.docx]

**Supplementary Figures**

**
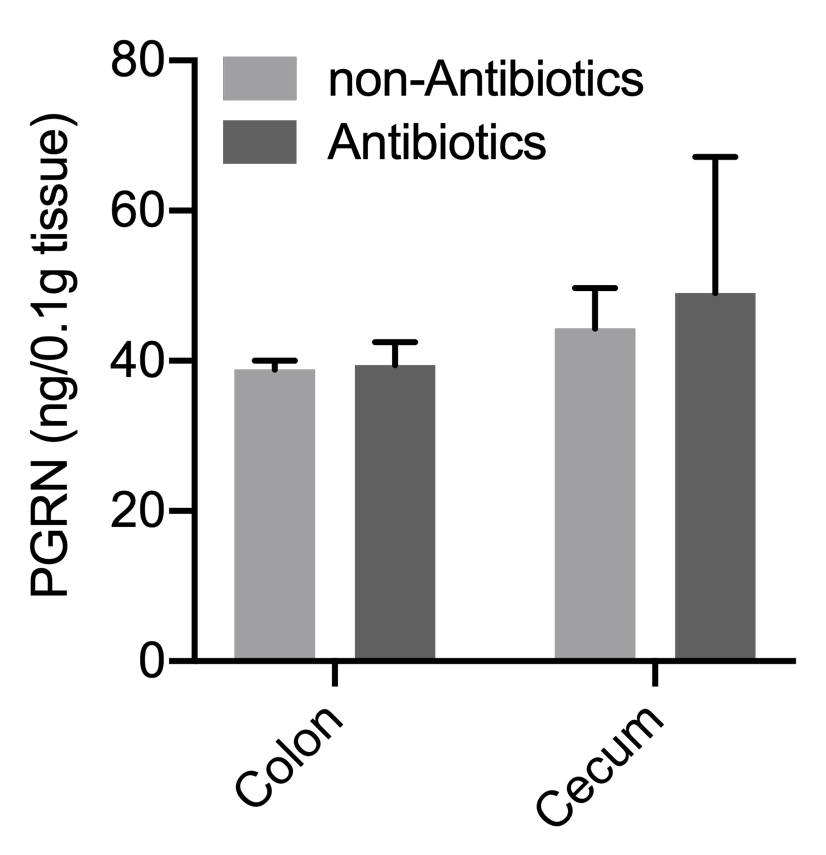
**

**Supplementary Figure 1. ﻿** **Antibiotic treatment had no effects on the production of PGRN in the colon and cecum of WT mice.** ELISA analysis of the PGRN protein levels in the colon and cecum of mice treated with or without antibiotics (n=9). Data were expressed as mean ± SD. Statistical significance was tested by two-tailed unpaired Student t-test.


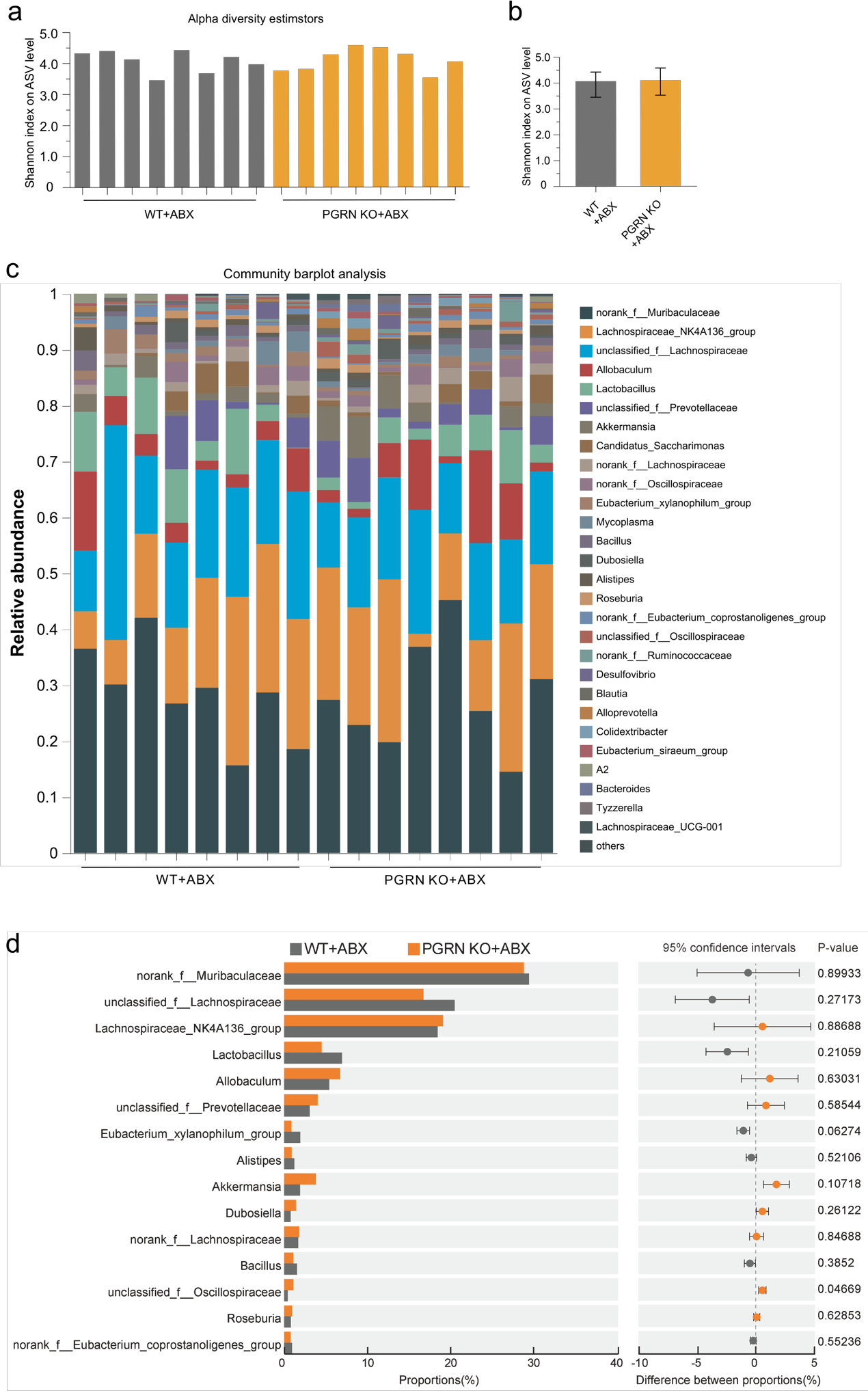


**Supplementary Figure 2. ABX WT and PGRN KO mice have no differences in microbiota diversity prior to CDI.** **(**a-b**)** Alpha diversity estimators calculated using indexes of Shannon on ASV level in ABX WT or PGRN KO mice prior to CDI (n=8). (c) Relative abundance of bacteria at the genus levels in ABX PGRN KO or WT mice prior to CDI (n=8). (d) Bacteria with no significant changes in the relative abundance at the genus level in ABX PGRN KO or WT mice prior to CDI (n=8). Data were expressed as mean ± SD (b). Statistical significance was tested by Wilcoxon rank-sum test (b and d).


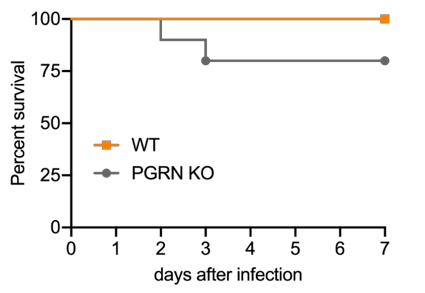


**Supplementary Figure 3. Survial in WT and PGRN KO mice upon *C.difficile* infection.** WT and PGRN KO mice were infected with a dose of 1×10^8^ CFUs *C. difficile* (VPI 10463) to established a mild CDI model, and survival was monitored (n=25-30). Survival curves were compared using a log rank statistical test.


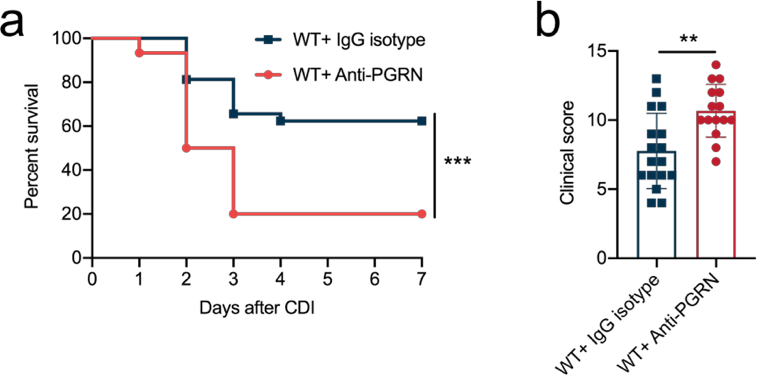


**Supplementary Figure 4. Mice treated with anti-PGRN antibodies had significantly increased mortality and clinical scores.** WT mice were orally infected by 1×10^9^ CFUs *C. difficile* and treated with isotype IgG control or anti-mouse PGRN antibodies (100 μg/mouse) on the indicated day. (a) Survival curves (n=30-32). (b) Clinical scores were assessed on day 2 (n=15-17). Survival curves were compared using a log rank statistical test (a). Data were expressed as mean ± SD. Statistical significance was tested by two-tailed unpaired Student t-test (b). ***p*< 0.01, ****p*< 0.001.


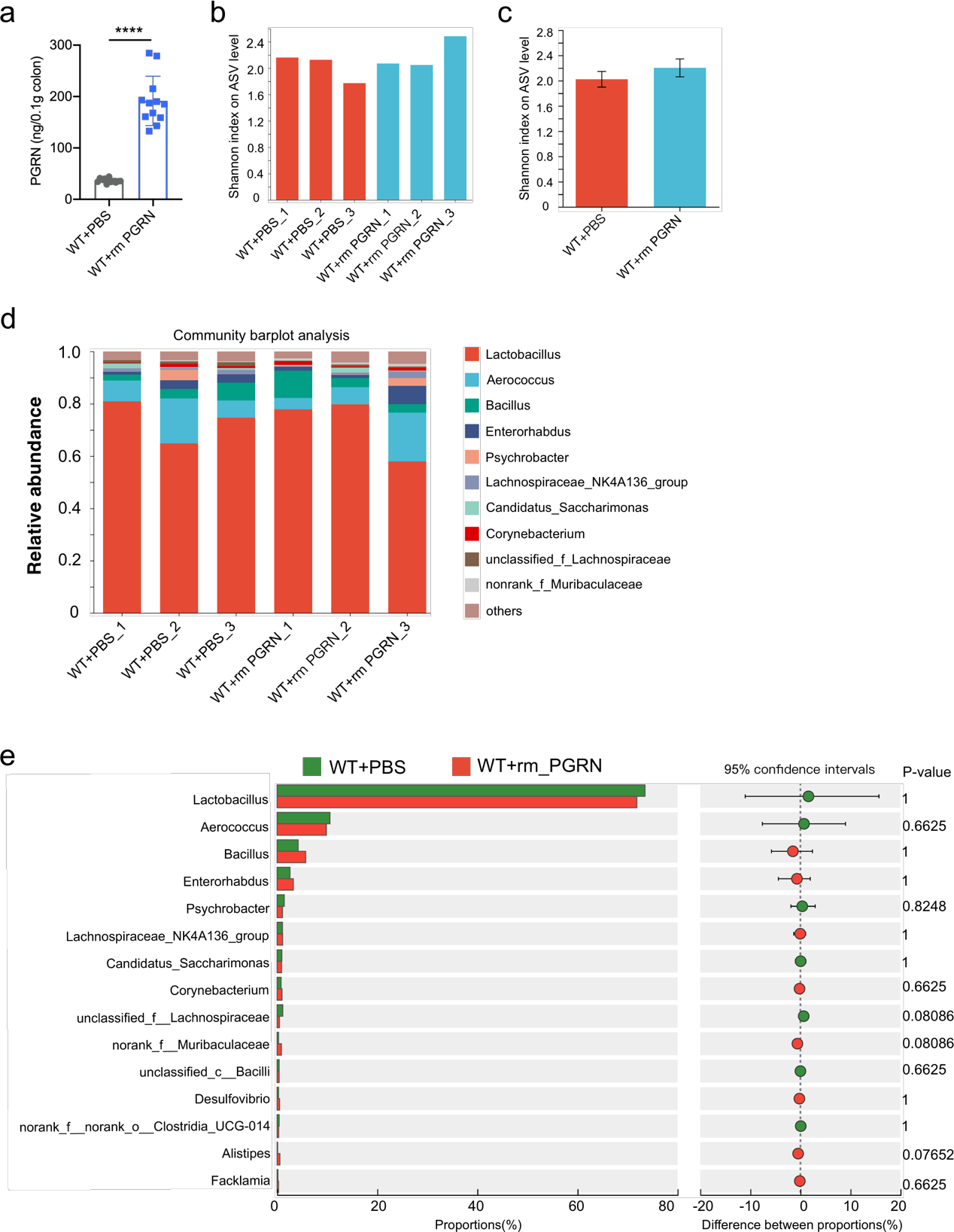


**Supplementary Figure 5. PGRN treatment did not significantly alter the microbiota composition**. WT mice were administrated with rm PGRN (10 μg/mouse) by intraperitoneal injection prior to CDI. (a) The expression of PGRN in colonic tissue were detected by ELISA (n=15). (b-c**)** Alpha diversity estimators calculated using indexes of Shannon on ASV level (n=3). (d) Relative abundance of bacteria at the genus levels (n=3). (e) Bacteria with no significant changes in the relative abundance at the genus level (n=3). Data were expressed as mean ± SD. Statistical significance was tested by two-tailed unpaired Student t-test (a). Statistical significance was tested by Wilcoxon rank-sum test (c and e). *****p<* 0.0001.


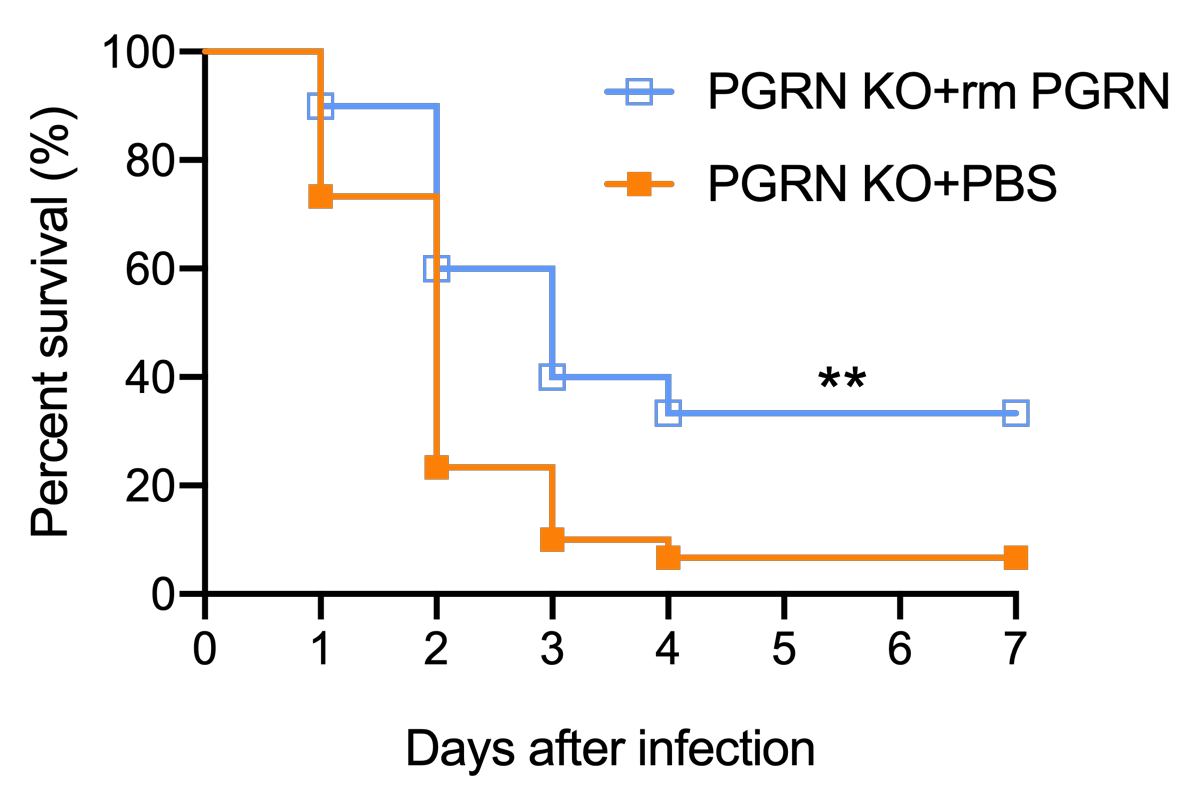


**Supplementary Figure 6.** **PGRN treatment enhanced protection against CDI in PGRN KO mice.** PGRN KO mice were injected intraperitoneally with 10 μg of recombinant mouse PGRN (rmPGRN) or vehicle control (PBS) after CDI, and survival was monitored (n = 30). Survival curves were compared using a log rank statistical test. ***p<* 0.01.

**
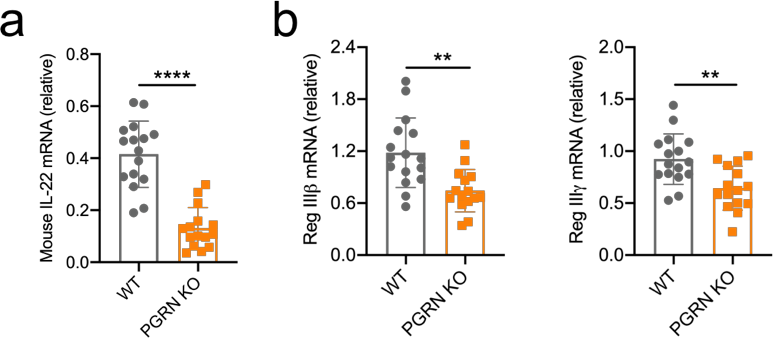
**

**Supplementary Figure 7. The mRNA expression levels of IL-22 and IL-22-associated downstream target genes in WT and PGRN KO mice after CDI.** WT and PGRN KO mice were orally infected with 1×10^9^ CFUs *C. difficile*. After infection, mice were sacrificed on day 2, and ﻿mRNA expression levels of (a) IL-22, (b) RegIIIβ and RegIIIγ of colonic tissuewere quantified (n=15-16), GAPDH was used as internal control. Data were expressed as mean ± SD. Statistical significance was tested by two-tailed unpaired Student t-test (a and b). ***p*< 0.01, *****p*< 0.0001.


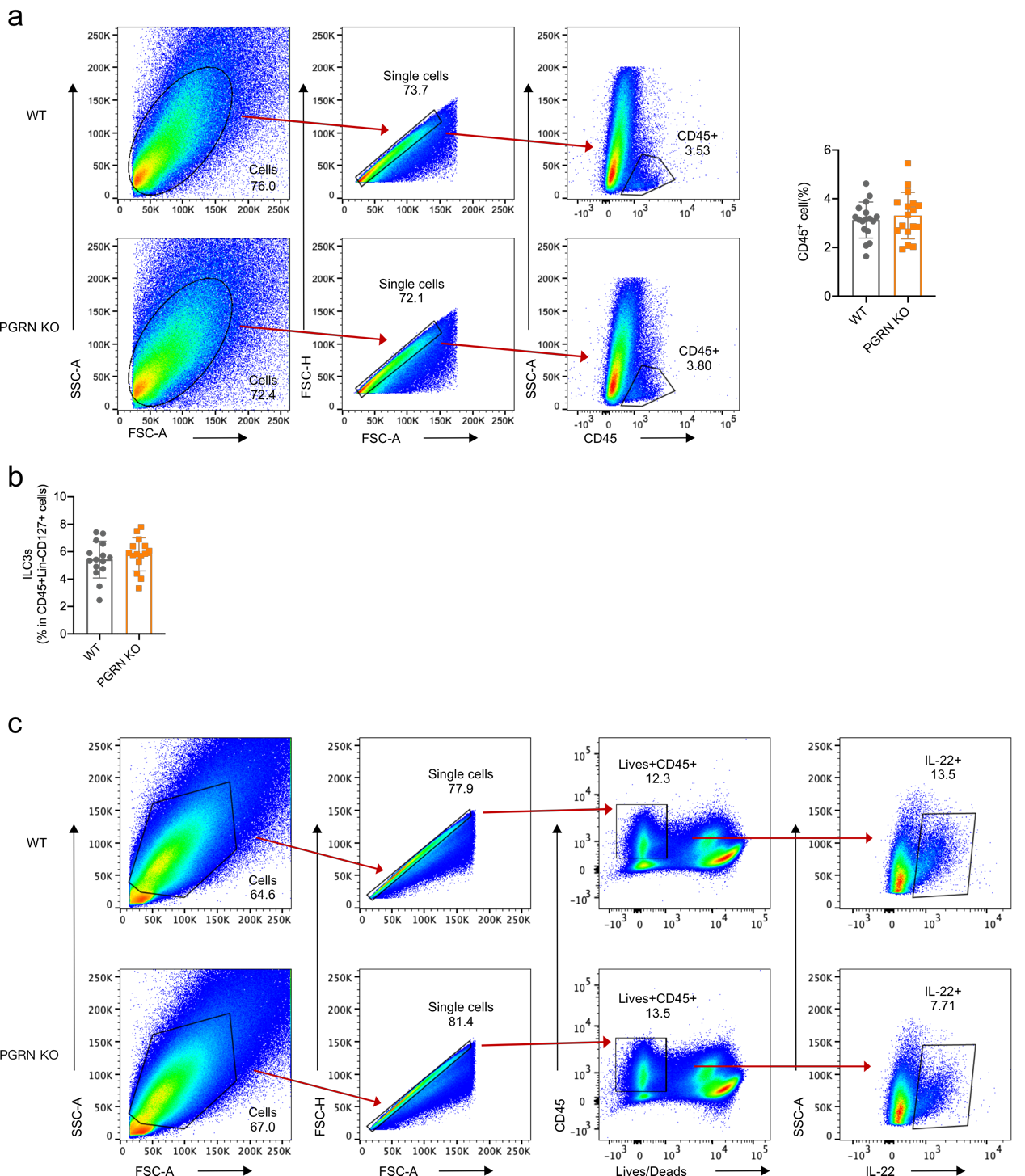


**Supplementary Figure 8.** **Gating strategy for flow cytometry.** WT and PGRN KO mice were orally infected by 1×10^9^ CFUs *C. difficile*. After infection, mice were sacrificed on day 2. (a) Gating strategy for flow cytometry of CD45^+^ cells (left) and the proportion (right) of CD45^+^ cells in the colonic lamina propria (n=17). (b) Abundance of ILC3 cells ( in CD45^+^Lineage^−^CD127^+^ cells) in the colonic lamina propria (n=15). (c) Gating strategy for flow cytometry of IL-22^+^CD45^+^ cells in the colonic lamina propria. Data were expressed as mean ± SD. Statistical significance was tested by two-tailed unpaired Student t-test (a and b).


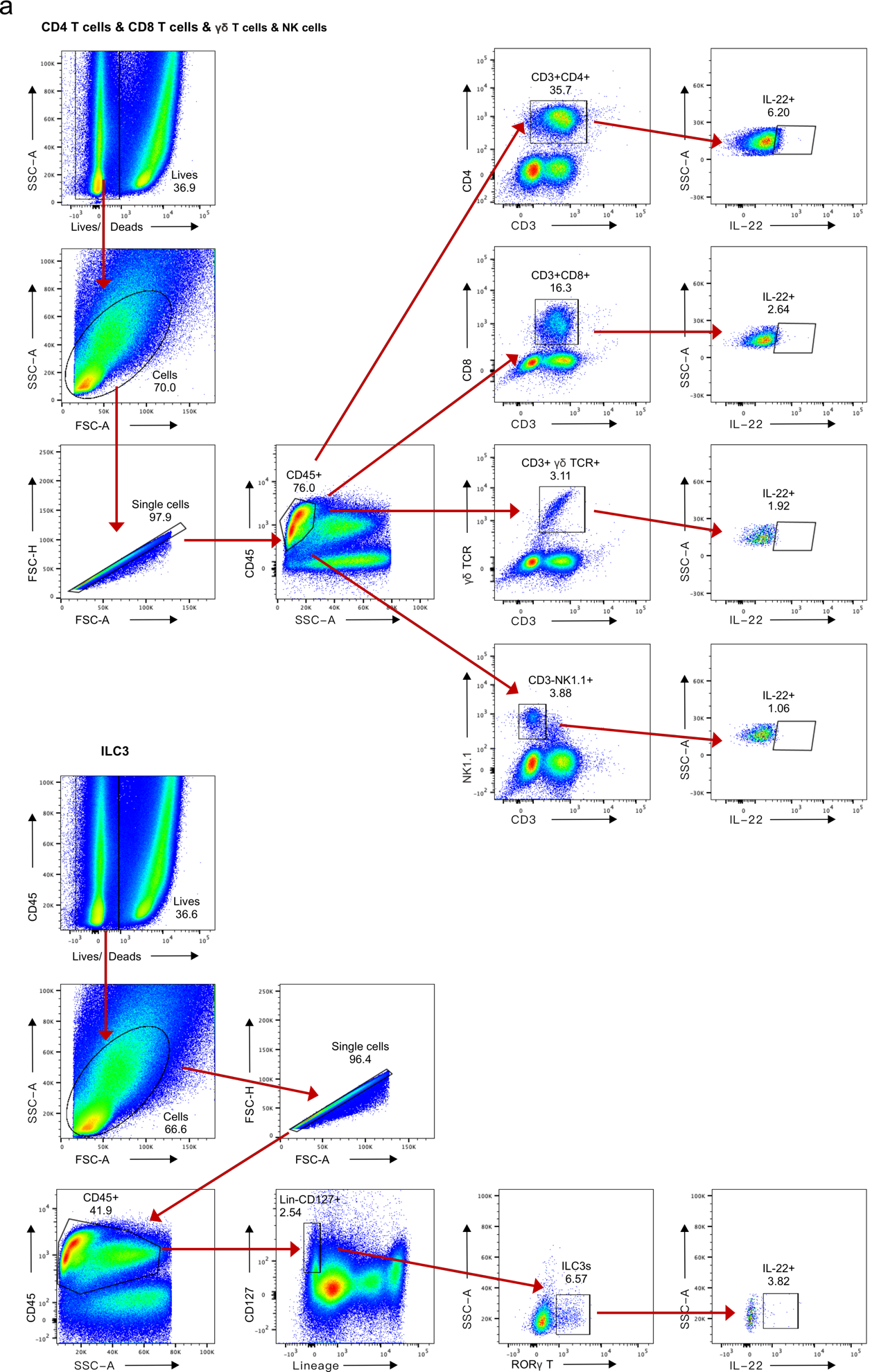


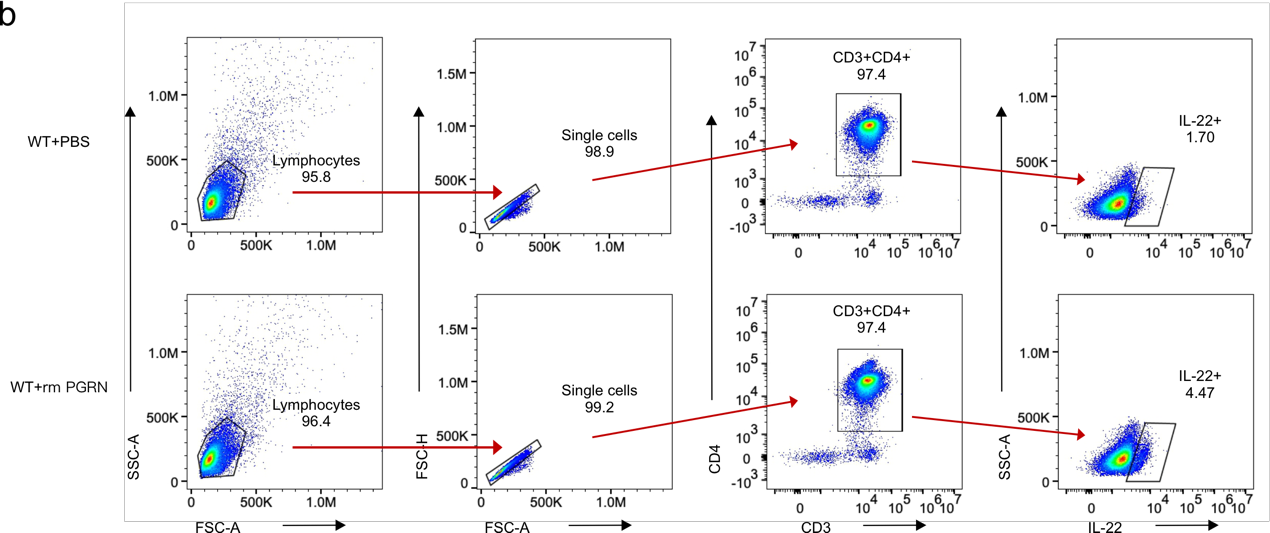


**Supplementary Figure 9.** **Gating strategy for flow cytometry.** (a) FACs gating strategy for IL-22^+^ cells in colonic CD4^+^ T helper cells, CD8^+^ T helper cells, γδ T cells, NK cells and ILC3. (b) FACs gating strategy for IL-22^+^ cells in mouse splenic CD4^+^ T cells.


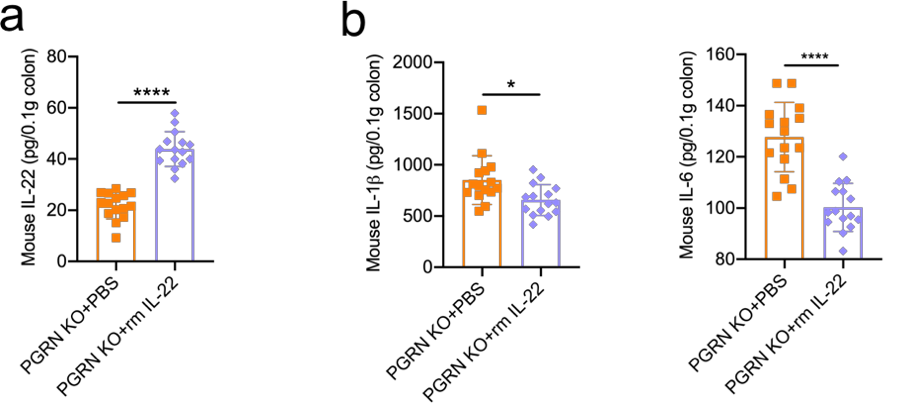


**Supplementary Figure 10. Restoration of IL-22 provided protection against CDI in PGRN KO mice.** (a) PGRN KO mice were intraperitoneally administrated with rm IL-22 (1 μg/mouse) prior to CDI and the colonic IL-22 expression levels were analyzed by ELISA (n=15). (b) PGRN KO mice were orally infected by 1×10^9^ CFUs *C. difficile* and treated with rmIL-22 (1 μg/mouse), and the expression levels of IL-1β and IL-6 in the colonic tissues at day 2 were measured by ELISA (n=15). Data were expressed as mean ± SD. Statistical significance was tested by two-tailed unpaired Student t-test. **p<* 0.05, *****p*< 0.0001.

**
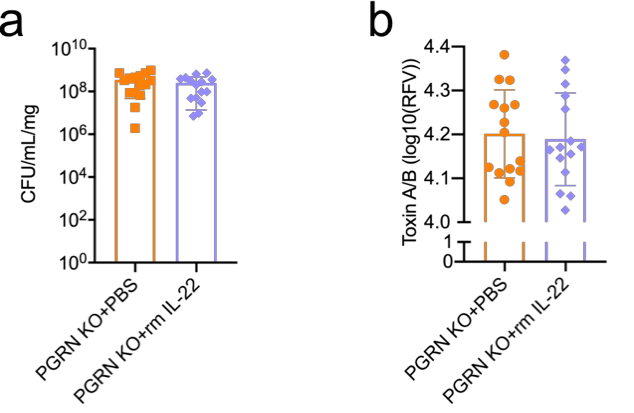
**

**Supplementary Figure 11. IL-22 had no effects on *C. difficile* bacterial burden and toxin A/B levels in PGRN KO mice.** PGRN KO mice were gavaged with 1×10^9^ CFUs *C. difficile* and treated with recombinant mouse IL-22 (rm IL-22, 1 μg/mouse) or vehicle control (PBS) by intraperitoneal injection. (a) *C. difficile* bacterial burden in cecal contents (n=15). (b) Toxin A/B in the cecal contents were assessed by VIDAS (n=15). Data were expressed as mean ± SD. Statistical significance was tested by two-tailed unpaired Student t-test.

**
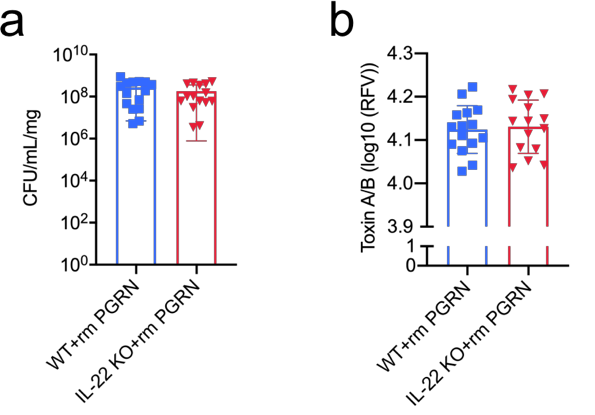
**

**Supplementary Figure 12. PGRN had no effects on *C. difficile* bacterial burden and toxin A/B levels in WT and IL-22 KO mice.** WT and IL-22 KO mice were infected by 1×10^9^ CFUs *C. difficile* and treated with rm PGRN intraperitoneally (10 μg/mouse). Mice were sacrificed on day 2. (a) *C. difficile* bacterial burden in cecal contents (n=15). (b) Toxin A/B in the cecal contents were assessed by VIDAS (n=15). Data were expressed as mean ± SD. Statistical significance was tested by two-tailed unpaired Student t-test.

**
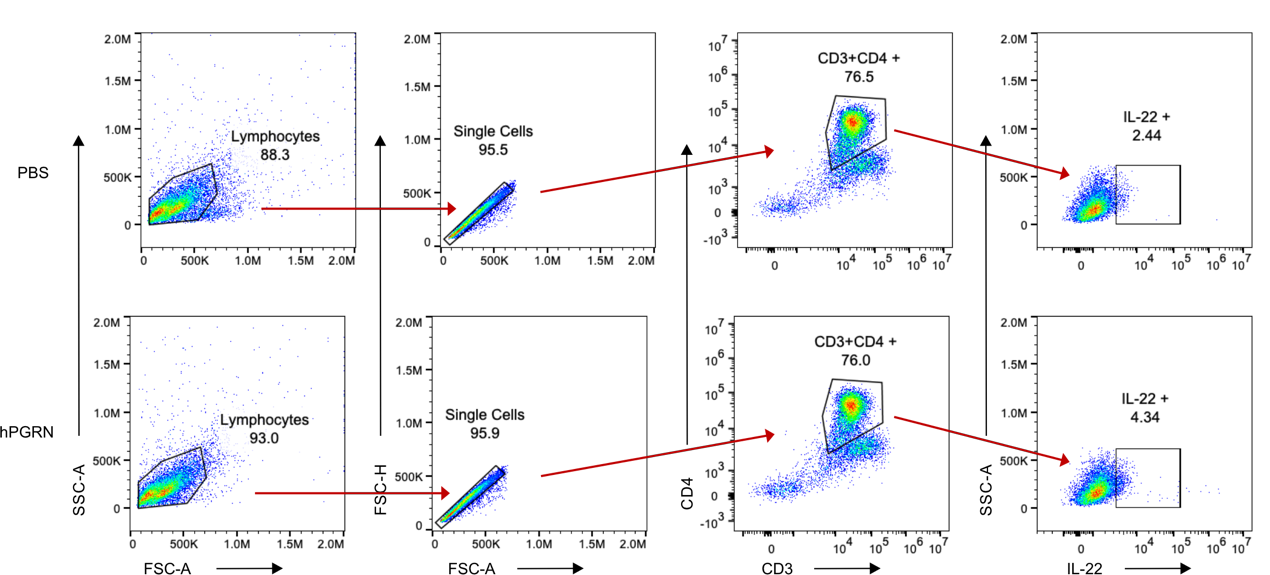
**

**Supplementary Figure 13. FACs gating strategy.** FACs gating strategy for IL-22^+^ cells in human peripheral blood CD4^+^ T cells.

**Supplementary Table**

| **Table S1. Baseline characteristics of the study patients and healthy controls** | | | |
| --- | --- | --- | --- |
| Variable | CDI-positive patients | CDI-negative patients | Healthy controls |
| Age(year) | 65(21-99) | 69.5（28-92） | 61(26-70) |
| Male/female | 40/40 | 18/14 | 18/17 |
| RBC, 10^12^/L | 3.45(1.52-7.75) | 3.83(2.07-4.92) | 4.62(4.1-5.67) |
| WBC, 10^9^/L | 7.55(0.02-29.24) | 6.11(1.99-15.93) | 5.48(3.9-8.41) |
| Neutrophil, 10^9^/L | 6.00(0-22.27) | 4.10(0.54-14.46) | 3.37(1.85-5.46) |
| Cr, μm/L | 57.5(13-625) | 66(38-482) | 64(48-84) |
| BUN，mm/L | 6.7(1.6-51) | 4.85(1.7-44.5) | 4.5(2.7-7.3) |
| ALB，g/L | 35(24-48) | 35(13-46) | 46(41-50) |
| CRP，mg/L | 29.7(2.5-225) | 16.4(0-221.1) | ND |
| PCT，ng/L | 0.3(0.01-88) | 0.21(0-2.98) | ND |
| RBC, red blood cells, WBC, white blood cells; Cr: creatinine; BUN: [blood urea nitrogen](https://www.baidu.com/link?url=ImKJuBsPnN69a3eKzWnY40_4v2nPo9_2Pei0lZ6-u0ArEMzikIdG3mLFdwGnciQjjvqghQ35y0BOQ0osezE_QMMe-XUjz5cDOSXWix3NTlPLmDJhs447S9NpUbG_5dfe&wd=&eqid=b6b93d1300002ca10000000559619554); ALB: albumin; CRP, C-reactive protein; PCT, procalcitonin; ND, not determined. Data are expressed as median (interquartile range). | | | |
